# NETosis Markers (ssDNA, dsDNA) as Predictors of Mortality and Hospitalization After Endovascular Aortic Repair

**DOI:** 10.3390/ijms27052427

**Published:** 2026-03-06

**Authors:** Milena N. Michalska, Tadeusz Grochowiecki, Aleksandra Wyczałkowska-Tomasik, Leszek Pączek, Michał Macech, Bartłomiej Antoń, Zbigniew Gałązka

**Affiliations:** 1 Department of General, Vascular, Endocrine and Transplant Surgery, Medical University of Warsaw, 04-749 Warsaw, Poland; tadeusz.grochowiecki@wum.edu.pl (T.G.); bartlomiej.anton@wum.edu.pl (B.A.); zbigniew.galazka@wum.edu.pl (Z.G.); 2 Department of Immunology, Transplantology, and Internal Diseases, Medical University of Warsaw, 04-749 Warsaw, Poland; a.tomasik@wum.edu.pl (A.W.-T.); leszek.paczek@wum.edu.pl (L.P.)

**Keywords:** neutrophils, extracellular neutrophils traps, aortic aneurysm

## Abstract

Neutrophils and their extracellular traps (NETs) are pivotal elements of the immune response. This study investigates the dynamics of neutrophil-related markers during the perioperative period of branched endovascular aortic repair (BEVAR) in patients with thoracoabdominal aortic aneurysms (TAAAs) and evaluates their association with one-year clinical outcomes. A prospective, single-center study was conducted on 20 TAAA patients treated with T-branch devices. The analysis focused on surrogate markers associated with NETosis, including double-stranded DNA (dsDNA), single-stranded DNA (ssDNA), and citrullinated histone H3 (citH3). Peripheral venous blood was collected 24 h before BEVAR, and on the third and fifth postoperative days. Patients were monitored for one year to evaluate mortality and hospitalization risks, with predictors identified using Cox regression analysis. Increased postoperative levels of inflammatory markers were significantly associated with higher risks of mortality and hospital readmission. On the third postoperative day, key parameters emerged as predictors of adverse outcomes: dsDNA (HR = 1.000; 95% CI 1.000–1.000; *p* = 0.027), ssDNA (HR = 1.000; 95% CI 1.000–1.000; *p* = 0.022), and NLR (HR = 1.226; 95% CI 1.043–1.440; *p* = 0.013). Markers assessed in the early postoperative period (the third postopearive day) demonstrated superior predictive utility compared to those measured on the fifth postoperative day. CitH3 levels did not show statistical significance as a prognostic factor. Early postoperative evaluation of NET-associated markers, particularly dsDNA and ssDNA, offers prognostic value for predicting mortality and hospitalization risks in TAAA patients undergoing BEVAR. These markers may provide superior predictive accuracy compared to conventional post-implantation syndrome criteria. Enhanced postoperative monitoring of these markers could help identify high-risk patients who may benefit from intensified follow-up.

## 1. Introduction

Endovascular aortic repair (EVAR) has grown in popularity in recent years based on the reduced risk of death and shorter recovery period observed compared to the risk of death and recovery period associated with open surgery in the treatment of thoracoabdominal aortic aneurysm (TAAA) [1]. However, there is presently no precise method for determining which patients are at increased risk of postoperative complications and mortality. Despite the benefits of endovascular treatment, accurate approaches for identifying high-risk individuals remain a significant clinical challenge. Age, female sex, dialysis, and the size and location of the aneurysm have all been linked to an elevated risk of postoperative mortality [2]. These characteristics, however, often lack sufficient predictive precision, and many individuals who do not display these risk factors may nonetheless develop complications following surgery. Recent studies have focused on the roles of neutrophils and neutrophil extracellular traps (NETs) in the pathophysiology of aortic aneurysms. Neutrophils are immune cells that aid in the inflammatory response. When neutrophils are stimulated, they can release NETs, which are neutrophil extracellular traps that can capture and destroy pathogens [3]. Excessive NET production, on the other hand, might cause tissue injury and inflammation. Neutrophils and NETs have been found in aortic aneurysm tissues, and their activity may lead to aneurysm development. Furthermore, after endovascular repair, an inflammatory state linked with neutrophilic activity has been reported [4]. Neutrophil activation and NET release have been described in detail in animal models of aortic aneurysm development, revealing the special role of neutrophils and neutrophil networks in the pathogenesis and progression of aortic aneurysm [5]. Neutrophils and NETs also play an indirect role in the development of aortic aneurysms by influencing other processes that contribute to the weakening of the aortic wall, such as atherosclerosis and chronic inflammation. Extracellular neutrophil networks can be assessed via evaluation of surrogate markers like double-stranded DNA (dsDNA), single-stranded DNA (ssDNA), and citrullinated histones H3 (CitH3) [6]. Despite the growing understanding of NETosis in vascular biology, its clinical utility in predicting long-term outcomes after complex endovascular repairs remains under-researched. The aim of this study was to assess the association of NET-associated markers, evaluated through surrogates such as dsDNA, ssDNA, and CitH3 based on mortality and hospitalization rates in patients with TAAA following endovascular treatment with T-branch devices during a one-year postoperative follow-up period.

## 2. Results

The study observed a cohort of 20 patients, with an average follow-up period of 314.68 ± 30.24 days (approximately 10.5 ± 1.00 months), corresponding to an interquartile range (IQR) of 289 to 399 days (or 9.63 to 11.30 months). During this time, two patients (10%) passed away. One death occurred 52 days postoperatively due to a hemorrhagic stroke while the patient was on dual antiplatelet therapy (DAPT). The second fatality, attributed to sudden cardiac arrest, happened 101 days after surgery. Table 1 provides detailed information on the medical events recorded throughout the observation period. Hospitalization was necessary for five patients (25%) during follow-up. The reasons included renal infarction caused by thrombosis, thrombosis in the right stent leg, anemia requiring blood transfusion, myocardial infarction, and paraplegia. Additionally, two patients (10%) were diagnosed with a type II endoleak that did not necessitate intervention. Stent thrombosis was reported in one patient (5%), occurring in the right femoral artery and requiring further treatment. Lower-limb dysfunction was observed in five patients (25%). Of these, three patients (15%) experienced intermittent claudication, and two patients (10%) developed paraplegia. Intermittent claudication emerged as a delayed symptom in all three cases, with two being temporary and one becoming permanent. Paraplegia presented immediately after surgery in one patient but resolved within four weeks. In the other case, paraplegia developed three weeks postoperatively and persisted until the end of the follow-up period. Dual antiplatelet therapy (DAPT) was recommended for three months post-surgery and was adhered to by 18 patients (90%). Beyond the recommended duration, three patients (15%) continued taking DAPT. Over the follow-up period, seven patients (35%) reached the study’s endpoint.

Significant perioperative variations were observed in the concentrations of all studied markers. The baseline median concentration of dsDNA in Collection I was 757.75 ng/mL (IQR: 520.75–1310.00). Following the procedure, a marked increase was noted in Collection II (median: 4907.50 ng/mL; mean: 5289.75 ± 2815.52 ng/mL), which remained elevated through Collection III (median: 4420.00 ng/mL). SsDNA concentrations followed a comparable trajectory, rising from a baseline median of 3625.00 ng/mL (IQR: 2212.50–7057.50) to 25,250.00 ng/mL in Collection II. Notably, ssDNA levels remained significantly higher than dsDNA levels at all time points, although the ssDNA/dsDNA ratio showed remarkable stability throughout the study period, with median values ranging from 4.47 to 4.65. The kinetics of Cit-H3 were characterized by a significant peak in the early postoperative phase (Collection II), with median levels reaching 49.23 ng/mL (Mean: 66.57 ± 54.10 ng/mL) compared to 16.67 ng/mL at baseline. By Collection III, Cit-H3 concentrations showed a downward trend toward preoperative values (Median: 27.73 ng/mL. Detailed concentrations are presented in Table 2.

The independent factors for endpoints (death and hospitalization) are the following parameters: dsDNA (in the second blood collection), ssDNA (in the second blood collection), neutrophils (in the second and third blood collections), and the ratio of neutrophils to lymphocytes (in the second and third blood collections). The results of the Cox hazard analysis of selected parameters is presented in Table 3.

## 3. Discussion

Neutrophils play a central role in mediating inflammation, making their activity a critical parameter in assessing surgical stress, wound healing, and the risk of postoperative complications, including sepsis [7]. Inflammatory processes are known to occur significantly after stent graft placement [8,9]. Their activation, particularly through the NETosis process, leads to the formation of neutrophil extracellular traps (NETs), which are structures composed of chromatin (including ssDNA and dsDNA) and neutrophil granule proteins.

At the molecular level, NETosis is triggered by intracellular signaling cascades including calcium influx, PKC activation, and NADPH oxidase-dependent reactive oxygen species (ROS) production, which promote nuclear membrane rupture. Neutrophil elastase (NE) and myeloperoxidase (MPO) translocate into the nucleus to degrade histones, while PAD4-mediated citrullination of histone H3 facilitates chromatin decondensation, enabling the extrusion of DNA and granule proteins into extracellular traps. Therefore, NETosis can be monitored in the blood using specific markers such as ssDNA, dsDNA, and Cit-H3 [10,11,12].

Despite their well-documented involvement in various inflammatory and thrombotic conditions, the role of NETosis in thoracoabdominal aortic aneurysm (TAAA) patients undergoing branched endovascular aneurysm repair (BEVAR) has remained largely unexplored. This study sought to address this gap by evaluating NETosis-related markers, including ssDNA, dsDNA, and Cit-H3, in relation to postoperative outcomes, such as mortality and hospitalization.

A significant postoperative increase in neutrophil activity was observed, with elevated levels of NETosis markers, particularly ssDNA and dsDNA, in Collection II (the third postoperative day). These markers emerged as key prognostic indicators, strongly correlating with the risk of death and rehospitalization within the first year after surgery. This postoperative neutrophil activation likely triggers NETosis via the ROS-mediated activation of NE and MPO, along with the PAD4-mediated citrullination of histone H3, leading to chromatin decondensation and the release of extracellular DNA.

Sustained ssDNA and dsDNA may promote thrombus formation by providing a scaffold for platelet adhesion and activating the intrinsic coagulation pathway via factor XII, while histones and proteases from NETs can induce endothelial damage, amplifying thrombo-inflammatory responses [13,14,15,16]. Notably, the Cit-H3 assay showed no significant effect on the survival analysis. This marker is detected in the bloodstream during the release of the neutrophil network in the initial period and is considered the most specific marker of NETosis because it is not produced by any other mechanism.

Cit-H3 specifically reflects PAD4-dependent histone citrullination during early NET release. Its transient presence in circulation can be explained by rapid clearance via nucleases and the transient window of acute neutrophil activation following surgical stress [17,18]. This may be further explained by its transient presence in the bloodstream, as its levels normalized by Collection III (the fifth postoperative day). In the case of other markers, such as ssDNA and dsDNA, their levels remain elevated, which indicates a persistent inflammatory process and the ongoing reconstruction of the NET network architecture. Persistent ssDNA and dsDNA may originate from apoptotic or necrotic vascular cells and from remodeling NETs, sustaining inflammatory signaling and facilitating ongoing thrombus architecture reconstruction [19,20].

It can be assumed that the persistently high concentration of cfDNA (dsDNA and ssDNA) until Collection III, when the concentration of Cit-H3 normalized, may have come from damaged cells that participated in the development of a thrombus between the stent and the artery wall. These NETs provide a molecular scaffold that captures platelets, erythrocytes, and coagulation factors such as factor XII and von Willebrand factor, promoting thrombus formation and stabilization between the stent and the vessel wall [21]. Platelets, erythrocytes, and coagulation factors are attached to the network of NETs, which are actively involved in thrombus formation. The process of remodeling the thrombus between the endothelium and the implanted stent allows for a reduction in the diameter of the aneurysm over a longer period of observation [22]. Remodeling of the thrombus is mediated by proteolytic activity from NE and MPO, which modulate the NET scaffold, and by thrombin activation within the NET matrix, contributing to fibrin deposition and gradual aneurysm sac remodeling [23].

The third postoperative day (Collection II) appears to be a critical time point for monitoring inflammatory and thrombotic responses. This day marked the peak of deviations in inflammation and NETosis-related parameters, highlighting its prognostic importance. These findings underscore the need for the regular monitoring of dsDNA and ssDNA levels during this period to identify patients at higher risk for adverse outcomes and guide early interventions. The peak in Collection II likely corresponds to maximal neutrophil activation at the molecular level, with ROS generation, PAD4-mediated histone citrullination, and NE/MPO activity reaching their highest levels, resulting in a peak cfDNA release and thrombo-inflammatory signaling [24,25].

While this study focused on NETosis markers, it also confirmed the prognostic value of the neutrophil-to-lymphocyte ratio (NLR) in assessing inflammation and predicting outcomes after BEVAR. At the molecular level, an increased NLR may reflect neutrophil activation via pro-inflammatory cytokines (e.g., IL-6, TNF-α) and ROS-mediated signaling, while lymphocyte counts decrease due to stress-induced apoptosis and suppression by anti-inflammatory mediators such as IL-10 and TGF-β [26,27]. NLR, a simple and cost-effective parameter, reflects the balance between neutrophil-driven inflammatory responses and lymphocyte-mediated immune regulation [28,29]. Neutrophils propagate inflammation through NET formation, ROS production, and protease release, while lymphocytes modulate this response via cytokines such as IFN-γ and IL-2, which regulate neutrophil activity and overall immune homeostasis [30,31].

An increase in neutrophil counts coupled with a decrease in lymphocyte counts leads to elevated NLR values, which are strongly associated with postoperative complications and survival. Elevated neutrophils contribute to tissue damage via NE and MPO, and cfDNA can further activate coagulation, whereas reduced lymphocyte-mediated regulation fails to restrain this inflammatory cascade, collectively increasing the risk of postoperative complications [32,33].

The data indicate that NLR peaks in Collection II (the third postoperative day), aligning with the highest inflammatory response. Elevated NLR values on this day were identified as independent predictors of mortality and hospitalization within one year post-surgery. The peak in Collection II likely corresponds to maximal neutrophil activation at the molecular level, characterized by ROS generation, NETosis, and cytokine release, while lymphocyte suppression through stress-induced apoptosis and anti-inflammatory cytokines reaches its apex [34,35].

Notably, preoperative NLR values (Collection I) did not show prognostic significance, emphasizing the importance of postoperative dynamics in inflammation monitoring. Preoperative NLR may not reflect underlying molecular stress or neutrophil activation because surgical trauma is required to trigger ROS generation, NETosis, and lymphocyte apoptosis [36]. The prognostic value of NLR in Collection II was superior to that observed in Collection III (the fifth postoperative day), further underscoring its importance as a timely marker for clinical decision-making. The third postoperative day represents the molecular peak of neutrophil activation, with maximal ROS production, PAD4-mediated NETosis, and NE/MPO activity, coupled with minimal lymphocyte-mediated immune regulation, explaining the superior prognostic value of NLR at this time point [37,38].

These findings are consistent with previous studies, which have reported that elevated NLR values are associated with worse outcomes in patients undergoing abdominal aortic aneurysm repair [39]. For instance, patients with an NLR greater than 5 were shown to have significantly higher 30-day mortality rates. The utility of NLR lies in its simplicity, as it requires only two routinely measured parameters, making it an accessible and efficient tool for postoperative risk stratification [40,41]. However, the absence of standardized cut-off values limits its widespread application, necessitating further research to establish universal thresholds for clinical use [42]. In this study, it was found that the NLR values in Collection II and Collection III are independent risk factors for death and hospitalization up to one year after thoracoabdominal aortic aneurysm surgery. It is worth noting that the NLR values in the preoperative period (Collection I) did not show significance in assessing the risk of death and hospitalization. It turned out that the determination of the NLR indicator in Collection II (the third postoperative day) has a greater prognostic value than in Collection III (the fifth postoperative day). Similar results were also observed for blood neutrophils and the percentage of neutrophils to leukocytes in Collection II and III, which clearly confirms the influence of these parameters on the risk of death and hospitalization.

There are studies that discuss the factors influencing short-term postoperative mortality in patients diagnosed with post-implantation syndrome (PIS). However, no differences have been observed in long-term observations. PIS is a poorly understood phenomenon that occurs in the early period after the endovascular repair of an aortic aneurysm. There are no unequivocal diagnostic symptoms, which makes it difficult to diagnose. Diagnostic criteria include leukocytosis and fever [43]. Due to these criteria, it is very important to differentiate between post-implantation syndrome and systemic inflammatory response syndrome (SIRS). PIS is considered a complication of endovascular surgery and is often observed within two days after surgery. The cause of the syndrome is complex and includes several sources at the cellular and organ levels [44]. It is reported that neutrophil degranulation observed after surgery may also be closely related, but only a few studies have included this phenomenon [45]. Short-term follow-up of patients diagnosed with PIS after endovascular treatment of aortic aneurysm suggested that these patients are at high postoperative and mortality risk [46]. Leukocytosis, with neutrophils being the predominant fraction, remains a significant criterion in the diagnostic profile of post-implantation syndrome.

The findings of this study emphasize the critical role of neutrophils and NETosis in the inflammatory and thrombotic responses following EVAR for TAAA. The sustained elevation of dsDNA and ssDNA levels reflects ongoing inflammatory activity and thrombus remodeling, making these markers valuable prognostic tools for assessing postoperative risk. Moreover, the NLR, as a simple yet robust indicator of inflammation, provides additional prognostic insights, particularly when measured on the third postoperative day (Collection II).

## 4. Materials and Methods

### 4.1. Research Project

This study is a prospective, single-center observational investigation conducted at a high-volume clinical center that performs over 150 endovascular aortic repair (EVAR) procedures for thoracoabdominal aortic aneurysms (TAAAs) annually. To ensure maximal cohort uniformity, the study group was restricted to patients undergoing treatment exclusively with T-Branch multibranched stent graft (Cook Medical, Bloomington, IN, USA).

The study protocol was developed in accordance with the Declaration of Helsinki and received formal approval from the Bioethics Committee of the Medical University of Warsaw (approval number: KB/168/2020). All participants provided written informed consent prior to enrollment. Inclusion and exclusion criteria are shown in Table 4.

### 4.2. Material

A total of 20 patients (85% male, 15% female) with thoracoabdominal aortic aneurysms (TAAAs) without identified wall rupture underwent endovascular repair using T-Branch multibranched stent graft. The mean age of the cohort was 71 ± 6 years. Regarding body mass index (BMI), 50% of the patients had a BMI > 25 kg/m^2^, of whom 35% were classified as obese (BMI > 30 kg/m^2^). The smoking history revealed that 60% of patients were former smokers with an average cessation period of 9 ± 7 years, while 35% were current smokers. Detailed demographic data are presented in Table 5.

The most prevalent comorbidities were hypertension (85%) and various cardiovascular diseases (65%). A history of myocardial infarction was reported in 25% of the cohort. All hypertensive patients (85%) were receiving pharmacological treatment, with beta-blockers being the most frequently prescribed (65%). Other therapies included diuretics (40%), calcium channel blockers (40%), ACE inhibitors (35%), and angiotensin receptor blockers (25%). Hyperlipidemia was present in 65% of patients, with 60% receiving lipid-lowering therapy, primarily statins (55%) and fibrates (10%). Notably, none of the patients in the study group were diagnosed with diabetes. Detailed comorbidity data are summarized in Table 6.

### 4.3. Endovascular Surgery of Thoracoabdominal Aortic Aneurysm

The surgeries were carried out by an experienced operator (TJ) in a hybrid operating room, utilizing both left axillary and femoral artery access points. The surgeries were performed under general anesthesia and after obtaining written informed consent from each patient. The primary indications for stent graft placement included a maximal aortic diameter of ≥55 mm or rapid aneurysm expansion defined as ≥10 mm within 12 months. A through-and-through guidewire was placed between the left axillary and right femoral access sites. Following initial angiographic imaging, the appropriate endografts were deployed, including the t-Branch stent (Cook Medical, Bloomington, IN, USA) in the visceral segment of the aorta. Bridging stents were inserted into the celiac trunk, superior mesenteric artery, and both primary renal arteries through the axillary access. Accessory renal arteries, when present, were embolized with coils. A final angiography was performed to ensure adequate blood flow to the lower extremities, internal iliac arteries, visceral branches, and stent graft components. Bridging-covered stents, such as BeGraft (Bentley, Innomed GmbH, Villingen-Schwenningen, Germany) and Fluency (Bard Peripheral Vascular, Tempe, AZ, USA), were used, with additional relining using Zilver stents (Cook Medical, Bloomington, IN, USA) as needed to optimize vessel patency.

### 4.4. Perioperative Pharmacotherapy

Patients received 1 g of the antibiotic cefazolin intravenously 30–60 min prior to endovascular surgery. All patients received intraoperative unfractionated heparin (UFH) infusion, the results of which were compared to a 200–250 s actual clotting time (ACT).

Postoperatively, all patients received UFH infusion at a dose confirmed by the activated partial thromboplastin time (APTT) (2–2.5× normal) for 2 days. Oral antiplatelet therapy—DAPT—(clopidogrel 75 mg and ASA 75 mg once a day) was started on the second postoperative day and successfully after discharge.

### 4.5. Blood Collection

Peripheral venous blood samples were drawn at three time points: 24 h prior to the EVAR procedure, three days postoperatively, and on the fifth postoperative day, which often coincided with the patient’s discharge from the hospital. Blood collection was performed aseptically from an antecubital vein using standard venipuncture techniques. To maintain consistency, all samples were obtained by the same individual, employing a gentle tourniquet that was promptly released. The collected samples were carefully mixed by gently inverting the tubes to minimize procedural variability. A total of five tubes were collected: four tubes containing EDTA-stabilized plasma (Sarstedt Monovette EDTA KE 2.6 mL, Numbercht, Germany) and one tube for serum (Sarstedt Monovette Serum Z 4.9 mL, Numbercht, Germany). The sequence of the blood collection is presented in Table 7.

### 4.6. Plasma for Infectious Investigation

One tube of 2.6 mL of blood on EDTA-stabilized plasma was centrifuged (FrontierTM, Ohaus, FC5718R, Frankfurt, Germany) for 20 min at 340× *g* at room temperature; then, the supernatant was collected with a Pasteur pipette (Sarstedt, 86.1171.010, Numbrecht, Germany), of which 0.5 mL was pipetted into 2 sterile tubes (Sarstedt, 72.694.006, Numbrecht, Germany).

### 4.7. Plasma for cf, dsDNA, and cf, ssDNA Investigation

Three tubes of 2.6 mL of EDTA-stabilized plasma were centrifuged for 10 min at 1600× *g* at 4 °C; then, the supernatant was gathered with a Pasteur pipette (Sarstedt, 86.1171.010, Numbrecht, Germany) and placed in a tube (Sarstedt, 62.558.20, Numbrecht, Germany) and centrifuged again for 10 min at 16,000× *g* at 4 °C, after which the supernatant was harvested with a Pasteur pipette (Sarstedt, 86.1171.010, Numbrecht, Germany) and pipetted 2.0 mL into two sterile tubes (Sarstedt, 72.694.006, Numbrecht, Germany).

### 4.8. Serum for Cit-H3 Investigation

Blood obtained on the clot was left at room temperature for at least 30 min before centrifugation at 85× *g* for 10 min at room temperature; then, the supernatant was collected using a Pasteur pipette (Sarstedt, 86.1171.010), of which 0.5 mL pipetted into two sterile tubes (Sarstedt, 72.694.006). The material was obtained and stored in a freezer (Eppendorf Cryocube F740hi, Humburg, Germany) at −80 °C until analysis.

### 4.9. Quantitative Assessment of Free Circulating Double-Stranded DNA (cf, dsDNA) and Single-Stranded DNA (cf, ssDNA)

The first step was the isolation of free circulating DNA (cfDNA), performed with the MagMAXTM Cell-Free DNA Isolation Kit from Applied Biosystems (Cat. No. A29319, Applied Biosystems, Austin, TX, USA). The material for the research was blood plasma. An amount of 4 mL of edetate plasma was used for isolation. Isolation was carried out following the manufacturer’s instructions. The next step was the quantification of double-stranded DNA (dsDNA) and single-stranded DNA (ssDNA). In a sample of the isolated cfDNA, the concentration of double-stranded DNA was measured. This was performed using the Qubit dsDNA HS Assay Kit (Cat. No. Q32851, Invitrogen, Carlsbad, CA, USA) and the Qubit ssDNA Assay Kit from Invitrogen (Cat. No. Q10212, Invitrogen, Carlsbad, CA, USA). The study was conducted in doublets. The final measurement was made on Qubit 4 Fluorometer from Invitrogen.

### 4.10. Determining the Concentration of Cit-H3

The Cit-H3 concentrations were determined using an enzyme immunoassay using the ELISA method. Cayman’s Citrullinated Histone H3 (Clone 11D3) (Cat. No. 501620, Cayman, Ann Arbor, MI, USA) ELISA kit was used in the investigation. Serum served as the research’s initial stage. The test was performed in doubles. The final measurement was placed at a wavelength of λ = 450 nm on a BioTek Power Wave XS spectrophotometer from Bio-Tek Instruments (Winooski, VT, USA). Calculation of the Cit-H3 concentration in the test tubes was performed with the KCjuniorWin computer program (version 1.41 KCjunior for Windowds, BioTek Instrumennts Inc., Winooski, VT, USA), using the standard curve method.

### 4.11. Observation of Patients After Endovascular Repair of Thoracoabdominal Aortic Aneurysm

One year after the surgery, the patient’s health status was monitored through a telephone interview. Information on the health status of all patients was collected, and the following events were assessed: death, myocardial infarction (MI), deep vein thrombosis (DVT), pulmonary embolism (PE), transient ischemic attack (TIA), stroke, stent narrowing, stent thrombosis, stent migration, aneurysm diameter enlargement, aortic wall dissection or rupture, endoleaks, reintervention, hospitalization, and lower limb disorders (paresthesia, paraplegia). A composite endpoint of death or hospitalization was defined for the evaluation of the predictive value of the studied parameters.

### 4.12. Statistical Analysis

Commercial software (Statsoft Statistica 13.3; Tibco Software, Palo Alto, CA, USA) was used to analyze the data. For every datum, descriptive statistics were supplied. Shapiro-Wilk tests were used to validate the normality assumption. Most sample parameter distributions were non-normal, and Levene’s tests revealed heteroscedasticity in these circumstances. As a result, non-parametric approaches were used for all samples. The impact of the investigated factors on endpoint incidence was determined using Cox hazard regression. A *p*-value < 0.05 was considered significant.

## 5. Conclusions

Our analysis demonstrated that the independent risk factors for death and hospitalization within one year of follow-up after BEVAR for TAAA are as follows: dsDNA and ssDNA (key NETosis markers), absolute neutrophil counts, and the neutrophil-to-lymphocyte ratio (NLR). The evaluation of these NETosis-related parameters provides critical insights into the inflammatory and thrombotic landscape, ultimately helping to determine the risk of serious postoperative complications in this high-risk patient population.

## Figures and Tables

**Table 1:** Details of medical events that occurred after surgery during the observation period.

Observation	No (%)
Mortality	2 (10%)
Re-hospitalization	5 (25%)
Myocardial infarction	1 (5%)
Stroke	1 (4%)
Stent thrombosis	1 (5%)
Endoleak	2 (10%)
Reintervention	1 (5%)
Claudication	3 (15%)
Paraplegia	2 (10%)

**Table 2:** Perioperative dynamics of NET-associated surrogate markers in the study group.

Marker	Collection I (Preoperative)	Collection II (III Day Postoperative)	Collection III (V Day Postoperative)
dsDNA [ng/mL]			
Mean ± SD	1347.93 ± 1551.65	5289.75 ± 2815.52	5340.50 ± 3767.26
Median (IQR)	757.75 (520.75–1310.00)	4907.50 (3500.00–6892.50)	4420.00 (2682.50–6677.50)
ssDNA [ng/mL]			
Mean ± SD	6551.00 ± 7987.49	26,151.75 ± 14,182.07	27,426.75 ± 18,367.58
Median (IQR)	3625.00 (2212.50–7057.50)	25,250.00 (14,225.00–35,975.00)	25,225.00 (13,650.00–36,325.00)
Cit-H3 [ng/mL]			
Mean ± SD	16.20 ± 9.92	66.57 ± 54.10	26.97 ± 17.48
Median (IQR)	16.67 (7.76–20.30)	49.23 (21.87–99.27)	27.73 (11.44–33.92)
ssDNA/dsDNA			
Mean ± SD	4.67 ± 0.79	4.93 ± 0.91	4.84 ± 0.62
Median (IQR)	4.47 (4.27–4.86)	4.65 (4.33–5.50)	4.55 (4.37–5.21)

**Table 3:** The Cox hazard analysis of selected parameters.

Parameter Evaluation	*p* Value	Hazard Ratio (HR)	95% CI HR Lower	95% CI HR Upper
I-dsDNA [ng/mL]	0.744	1.000	1.000	1.001
II-dsDNA [ng/mL]	0.027	1.00026	1.000029	1.00049
III-dsDNA [ng/mL]	0.082	1.000	1.000	1.000
I-ssDNA [ng/mL]	0.514	1.000	1.000	1.000
II-ssDNA [ng/mL]	0.022	1.00006	1.000009	1.00011
III-ssDNA [ng/mL]	0.110	1.000	1.000	1.000
I-CitH3 [ng/mL]	0.743	1.013	0.936	1.098
II-CitH3 [ng/mL]	0.328	1.006	0.994	1.018
III-CitH3 [ng/mL]	0.884	1.003	0.964	1.044
I-NEU [10^3^/µL]	0.522	0.855	0.529	1.381
II-NEU [10^3^/µL]	0.028	1.327	1.030	1.709
III-NEU [10^3^/µL]	0.045	1.259	1.005	1.579
I-WBC [10^3^/µL]	0.529	0.899	0.646	1.252
II-WBC [10^3^/µL]	0.310	1.108	0.909	1.350
III-WBC [10^3^/µL]	0.331	1.113	0.897	1.381
I-LYM [10^3^/µL]	0.691	0.898	0.527	1.530
II-LYM [10^3^/µL]	0.446	0.633	0.195	2.053
III-LYM [10^3^/µL]	0.230	0.484	0.148	1.582
I-neu/limf ratio	0.861	0.958	0.593	1.547
II-neu/limf ratio	0.013	1.226	1.043	1.440
III-neu/limf ratio	0.016	1.179	1.031	1.347
I-NEU [%]	0.494	1.019	0.966	1.075
II-NEU [%]	0.012	1.149	1.031	1.283
III-NEU [%]	0.022	1.107	1.015	1.207
I-LYM [%]	0.828	0.994	0.939	1.052
II-LYM [%]	0.052	0.852	0.725	1.001
III-LYM [%]	0.197	0.930	0.833	1.038

Note: Statistically significant parameters (*p* < 0.05) are highlighted in red.

**Table 4:** Inclusion and exclusion criteria.

Inclusion Criteria:	Exclusion Criteria:
1. Patients diagnosed with TAAA 2. Indication for endovascular aortic repair using t-Branch stent 3. Age > 18 years 4. Written informed consent to participate in the study	1. Coexisting severe malignancy 2. Coexisting viral or bacterial infection 3. Patients with other diseases causing advanced organ failure: respiratory/cardiovascular/renal/liver 4. Pregnant women 5. Patients in poor general condition (>3 on ECOG/WHO scale)

**Table 5:** The patients’ characteristics.

Variable	Mean ± SD; Median (IQR) or No (%)
Age [years]	71 ± 6; 71.50 (65.50–75.00)
BMI [kg/m^2^]	26.82 ± 4.68; 24.89 (23.18–30.95)
BMI > 25	10 (50%)
BMI > 30	7 (35%)
Sex Female	3 (15%)
Male	17 (85%)
Smoker status Never	1 (5%)
Past	12 (60%)
Time without smoking (years)	9 ± 7; 5.00 (5–20)
Current	7 (35%)
Pack-year [years]	29 ± 17; 27.00 (17.50–40)

**Table 6:** Comorbidities and their frequencies.

Comorbidities	No (%)
Cardiological diseases	13 (65%)
Including only myocardial infarction	5 (25%)
Hypertensive	17 (85%)
Including only hypertensive therapy	17 (85%)
Hyperlipidemia	13 (65%)
Including only lipid-lower therapy	12 (60%)
Pulmonary diseases	7 (35%)
Including only COPD	3 (15%)
Neurological diseases	3 (15%)
Including only stroke	2 (10%)

**Table 7:** The sequence of the blood collection.

**Preoperative period**	Collection I	−24 h
**Postoperative period**	Collection II	3rd day after surgery
	Collection III	5th day after surgery

## Data Availability

The data presented in this study are available on request from the corresponding author. The data are not publicly available due to privacy and ethical restrictions.
